# Gas-Phase Reaction of *trans*-2-Methyl-2-butenal with Cl: Kinetics, Gaseous Products, and SOA Formation

**DOI:** 10.3390/atmos11070715

**Published:** 2020-07-05

**Authors:** María Antiñolo, María Asensio, José Albaladejo, Elena Jiménez

**Affiliations:** 1 Instituto de Investigación en Combustión y Contaminación Atmosférica, Universidad de Castilla-La Mancha, Camino de Moledores s/n, 13071 Ciudad Real, Spain; 2 Departamento de Química Física, Universidad de Castilla-La Mancha, Avda, Camilo José Cela 1B, 13071 Ciudad Real, Spain

**Keywords:** Cl atom, branched unsaturated aldehyde, kinetics, gas-phase products, secondary organic aerosol (SOA)

## Abstract

The gas-phase reaction between *trans*-2-methyl-2-butenal and chlorine (Cl) atoms has been studied in a simulation chamber at 298 ± 2 K and 760 ± 5 Torr of air under free-NO_x_ conditions. The rate coefficient of this reaction was determined as *k* = (2.45 ± 0.32) × 10-^10^ cm^3^ molecule^−1^ s^−1^ by using a relative method and Fourier transform infrared spectroscopy. In addition to this technique, gas chromatography coupled to mass spectrometry and proton transfer time-of-flight mass spectrometry were used to detect and monitor the time evolution of the gas-phase reaction products. The major primary reaction product from the addition of Cl to the C-3 of *trans*-2-methyl-2-butenal was 3-chloro-2-butanone, with a molar yield (Y_Prod_) of (52.5 ± 7.3)%. Acetaldehyde (Y = (40.8 ± 0.6)%) and HCl were also identified, indicating that the H-abstraction by Cl from the aldehyde group is a reaction pathway as well. Secondary organic aerosol (SOA) formation was investigated by using a fast mobility particle sizer spectrometer. The SOA yield in the Cl + *trans*-2-methyl-2-butenal reaction is reported to be lower than 2.4%, thus its impact can be considered negligible. The atmospheric importance of the titled reaction is similar to the corresponding OH reaction in areas with high Cl concentration.

## 1 Introduction

Carbonyl compounds are volatile organic compounds (VOCs) emitted into the troposphere by several natural sources and by on-road traffic [1–3]. In addition, they can be generated through the oxidation of other VOCs present in the troposphere [4]. For example, unsaturated carbonyls, such as *trans*-2-methyl-2-butenal (*E*-CH_3_CH=C(CH_3_)C(O)H), are emitted into the lower atmosphere from vegetation and human activities such as road vehicles and industries, and methylbutenals have been proposed as oxidation products of isoprene initiated by hydroxyl (OH) radicals in the presence of NO [5,6]. *Trans*-2-methyl-2-butenal can also be found in food items such as herbs and spices, tomatoes, animal foods, and spearmint, and it is a building block in organic synthesis [7].

Recently, there have been multiple studies on the gas-phase reactivity of unsaturated oxygenated compounds with tropospheric oxidants such as OH radicals, nitrate (NO_3_) radicals, or ozone (O_3_), but kinetic data on the reaction initiated by chlorine (Cl) atoms are scarce [8]. Some examples are the results previously published on the Cl reactivity of linear unsaturated aldehydes [9,10], methacrolein [11], or 3-methyl-2-butenal [12]. The gas-phase kinetics of *trans*-2-methyl-2-butenal was experimentally studied with OH radicals and O_3_ [13,14] and an estimation was reported for the NO_3_ and OH reactions by using Structure-Reactivity and Linear Free-Energy Relationships [15]. In contrast, there is no investigation or estimation of the rate coefficient for the reaction of *trans*-2-methyl-2-butenal with Cl or the products that are generated in reaction (R1).

(R1) $$E-{\text{CH}}_{3}\text{CH}=\text{C}\left({\text{CH}}_{3}\right)\text{C}(\text{O})\text{H}+\text{Cl}\to \text{Products.}$$

As aldehydes are known to be important contributors to the formation of secondary organic aerosols (SOA) [16,17], it is expected that SOA could be produced during the tropospheric oxidation of *trans*-2-methyl-2-butenal by Cl atoms. For the OH reaction, a SOA yield of 39.1% was determined by Chan et al. [18].

The aim of this research is, firstly, to determine the rate coefficient, *k*, of the gas-phase reaction (R1) at 298 ± 2 K and 760 ± 5 Torr under free-NO_x_ conditions by using two atmospheric simulation chambers. In the kinetic study, a relative method has been employed, using Fourier transform infrared spectroscopy (FTIR) as quantification technique. Secondly, in a separate set of experiments, the gaseous products of reaction (R1) have been detected by three different techniques: FTIR, gas chromatography coupled to mass spectrometry (GC-MS), and proton transfer time-of-flight mass spectrometry (PTR-ToF-MS). The formation of SOA has been monitored as a function of the reaction time by a Fast Mobility Particle Sizer (FMPS) spectrometer. A reaction mechanism is proposed based on all the identified products. Finally, a discussion on the impact of reaction (R1) in coastal clean environments, where there are high Cl and low NO_x_ concentrations is presented.

## 2 Experiments

The kinetic and product studies were performed in two atmospheric simulation chambers that were already described in previous studies [19,20]: a 16-L gas cell and a 264-L chamber. The 16-L gas cell is made of Pyrex and is a White-type cell with three mirrors inside and an optical path length of 96 m. The 264-L chamber is a cylindrical chamber made of Pyrex with stainless steel covers at the flat parts. Both chambers are surrounded by actinic lamps (Philips Actinic BL TL 40W/10 1SL/25, **λ** = 340–400 nm): four for the 16-L gas cell and eight for the 264-L chamber. These lamps were used to generate Cl atoms in situ by photolysis of Cl_2_. Gas-phase species were introduced from a gas-line with two capacitor pressure transducers (Leybold, model Ceravac, 10 and 1000 Torr full scale). Coupled to the chambers and depending on the experiment performed, specific instrumentation was used to monitor the time evolution of gaseous and particulate species. All the experiments were run by using synthetic air as bath gas at 760 ± 5 Torr and 298 ± 2 K.

### 2.1 Kinetic Study

For the kinetic experiments, mixtures of *trans*-2-methyl-2-butenal (T2M2B, henceforth), a reference compound (Ref, cyclohexane or isoprene), and Cl2 were introduced in the 16-L cell. Ranges of the initial concentrations in the cell were [T2M2B]_0_ = (3.4–7.1) × 10^14^ molecule cm^−3^, [cyclohexane]_0_ = (2.9–5.5) × 10^14^ molecule cm^−3^ or [isoprene]_0_ = (4.6–6.4) × 10^14^ molecule cm^−3^, and [Cl_2_]_0_ = (4.7–10.4) × 10^14^ molecule cm^−3^. In the performed experiments, T2M2B and the reference compound mainly react with Cl with rate coefficients *k* and k_Ref_, respectively, although they can also be lost by heterogeneous reaction onto the walls, photo induced processes, and/or reaction with the oxidant precursor. The additional loss is described by the first-order rate coefficients k_loss_ and k_Ref,loss_ that were evaluated in preliminary experiments in which the decay of the compound was monitored without Cl precursor in dark conditions and with the lamps switched on, and in the presence of Cl_2_ in the dark, as described in previous studies [19,20]. Considering the overall losses for T2M2B and Ref, the integrated rate equation is described by the following equation:

(1) $$\text{ln}\left(\frac{{\left[\text{T}2\text{M}2\text{B}\right]}_{0}}{{\left[\text{T}2\text{M}2\text{B}\right]}_{\text{t}}}\right)-k_{\text{loss}}t=\frac{k}{k_{\text{Ref}}}\left[\text{ln}\left(\frac{{\left[\text{Ref}\right]}_{0}}{{\left[\text{Ref}\right]}_{\text{t}}}\right)-k_{\text{Ref},\text{loss}}t\right],$$

where the subscript 0 indicates the initial concentrations of T2M2B and the reference compound and the subscript *t* indicates those concentrations at a reaction time *t*. These concentrations were monitored by a FTIR spectrometer (Thermo Fisher Scientific, model Nicolet Nexus 870, Madison, WI, USA) coupled to the 16-L cell that has a liquid N_2_-cooled MCT (Mercury Cadmium Telluride) detector. IR spectra were recorded between 650 and 4000 cm^−1^ at a resolution of 2 cm^−1^, after the accumulation of 32 interferograms, every 2 min. The IR bands selected for monitoring the loss of T2M2B and the reference compounds were 1710–2710 cm^−1^ for T2M2B, 2880 cm^−1^ for cyclohexane, and 860–950 cm^−1^ for isoprene.

### 2.2 Gaseous Products Study

For the determination of the gaseous products of reaction (R1), the two simulation chambers were used depending on the employed detection technique: FTIR spectroscopy, CG-MS, or PTR-ToF-MS.

When products were detected by FTIR spectroscopy, the 16-L cell and the same instrumentation used in the kinetic experiments described above were used again [19,20]. Experiments were run by irradiating the air mixture with T2M2B ((2.3–5.3) × 10^14^ molecule cm^−3^) and Cl_2_ ((6.0–7.7) × 10^14^ molecule cm^−3^) in the cell.

A GC-MS system (Thermo Electron, models Trace GC Ultra and DSQ II, Milan, Italy) and a PTR-ToF-MS (IONICON, model PTR-TOF 4000, Innsbruck, Austria) were coupled to the 264-L chamber, but in independent experiments. The GC-MS was equipped with a BPX35 column (30 m × 0.25 mm ID × 0.25 μm, SGE Analytical Science, Milton Keynes, UK) [19] working at a temperature ramp that ranged between 40 and 250 °C. The Solid-Phase Microextraction (SPME) was used as sampling technique with a 50/30 μm divinylbenzene/carboxen/polydimethylsiloxane (DVB/CAR/PDMS) fiber (Supelco) that was exposed to the gas mixture in the chamber during 5 min. In this case, the initial concentrations of T2M2B and Cl_2_ ranged between 5.4 and 8.6 × 10^14^ molecule cm^−3^ and between 5.1 and 8.4 × 10^14^ molecule cm^−3^, respectively.

The PTR-ToF-MS employs H3O^+^ as the primary ion in the proton transfer procedure. The mass spectra of the reactive gas mixture were recorded with a time resolution of 20 s and the detected mass range was set between 29 and 390.86 amu. The E/N factor of the instrument was set at 137 Td and products were quantified by using the transmission calibration of the instrument. Since this instrument is more sensitive than the FTIR spectrometer and the SPME/GC-MS system, to avoid saturation of the intensity, the initial concentration of T2M2B and Cl_2_ in the chamber was reduced with respect to that employed in the FTIR and GC-MS experiments: (1.9–2.1) × 10^13^ molecule cm^−3^ for T2M2B and (2.3–2.4) × 10^13^ molecule cm^−3^ for Cl_2_. In addition, to get intensity signals below the saturation limit, the gas sample had to be diluted with an air flow at the inlet of the PTR-ToF-MS by means of a dynamic inlet dilution system. A dilution factor of 1/5 was enough to not saturate the intensity signal. This dilution factor was achieved taking into account that the fixed flow going into the reaction chamber of the PTR-ToF-MS is 80 sccm (standard cubic centimeter per minute), and setting the additional inlet flow at 70 sccm, so the total flow going through the inlet was 150 sccm. Since the dilution flow was set at 120 sccm, only 30 sccm of the total 150 sccm going through the inlet were sampled from the 264-L chamber.

In all the experiments, the products of the reaction of T2M2B with Cl were monitored during 60 min, and tests were done to establish the products generated during the UV light exposure of T2M2B and by its reaction with Cl_2_ in the dark. The product yield of a product (Y_Prod_) was determined as the ratio of its concentration, [Prod], and the loss of reactant, Δ[T2M2B]:

(2) $${\text{Y}}_{\text{Prod}}=\frac{\left[\text{Prod}\right]}{\Delta \left[\text{T}2\text{M}2\text{B}\right]}.$$

### 2.3 SOA Formation Study

As the quantification of SOAs formed in reaction (R1) requires the knowledge of the loss of T2M2B by reaction with Cl atoms, the experiments were performed simultaneously in the 16-L and 264-L chambers, which were connected by a Teflon tube. Thus, the FMPS spectrometer (TSI Incorporated, model 3091, Shoreview (MN), USA) sampled from the 264-L chamber and its outlet flow was brought to the 16-L cell. With this configuration, the gas mixture was continuously flowing through the two chambers, and data of the particles in the 5.6–560 nm range inside the chambers could be recorded by the FMPS every 1 s (although they were averaged for 1 min), and gaseous T2M2B could be monitored at the same time with the FTIR spectrometer every 2 min. The total timescale of the experiment was 80 min. In the first 10 min, the Cl_2_/T2M2B/air mixture was not irradiated (i.e., no Cl reaction was taking place) to monitor the dark losses of gaseous T2M2B or its reaction with Cl_2_. After that, the lamps were turned on and the Cl reaction started, monitoring T2M2B loss and particles during 60 min. To evaluate the loss of the formed SOAs by wall loss or through the filters of the FMPS, the lamps were switched off for 20 min more. At each reaction time, the concentration of T2M2B and the SOA mass were corrected by accounting for their loss as described in a previous research. [20] The SOA yield was determined as the ratio between the aerosol mass formed, M_SOA_, and the loss of reactant due to the Cl reaction, Δ[T2M2B]:

(3) $${\text{Y}}_{\text{SOA}}=\frac{{\text{M}}_{\text{SOA}}}{\Delta \left[\text{T}2\text{M}2\text{B}\right]}.$$

### 2.4 Chemicals

Gases: Synthetic air (Air Liquide, 99.999%) and Cl_2_ (Sigma Aldrich, 99.8%) were used as supplied. Liquids from Sigma Aldrich, with purities in brackets, were used after freeze-pump-thaw cycles: *trans*-2-methyl-2-butenal (96%), cyclohexane (99.9%), and isoprene (99%).

## 3 Results

This section includes the experimental results for the kinetic and product studies for reaction (R1), presenting the measurement of the Cl-rate coefficient, the identification of products both in the gas and particle phases, and the determination of the SOA yield.

### 3.1 Determination of the Rate Coefficient

In the preliminary experiments performed in the absence of UV light and/or in the absence of Cl_2_, different loss processes for T2M2B (*k*_loss_) and the two reference compounds (*k*_ref,loss_) were investigated and considered in the kinetic data analysis. The pseudo-first order rate coefficient in this case is given by

(4) $$k_{\text{loss}}=k_{\text{w}}+k_{\text{hν}}+k_{{\text{Cl}}_{2}}[{\text{Cl}}_{2}].$$

Wall losses (*k_w_*) for T2M2B and the two reference compounds were observed, whereas UV photo induced processes (k_hν_) were perceptible only for T2M2B, and the dark reaction with Cl_2_ (*k*_Cl_2__) was detected only for isoprene. *k*_w_, *k*_hν_, and *k*_Cl_2__ were quantified in each case and are summarized for the three compounds in Table 1. These losses contributed around 1–10% to the total decay of T2M2B and the reference compounds.

**Figure 1:**
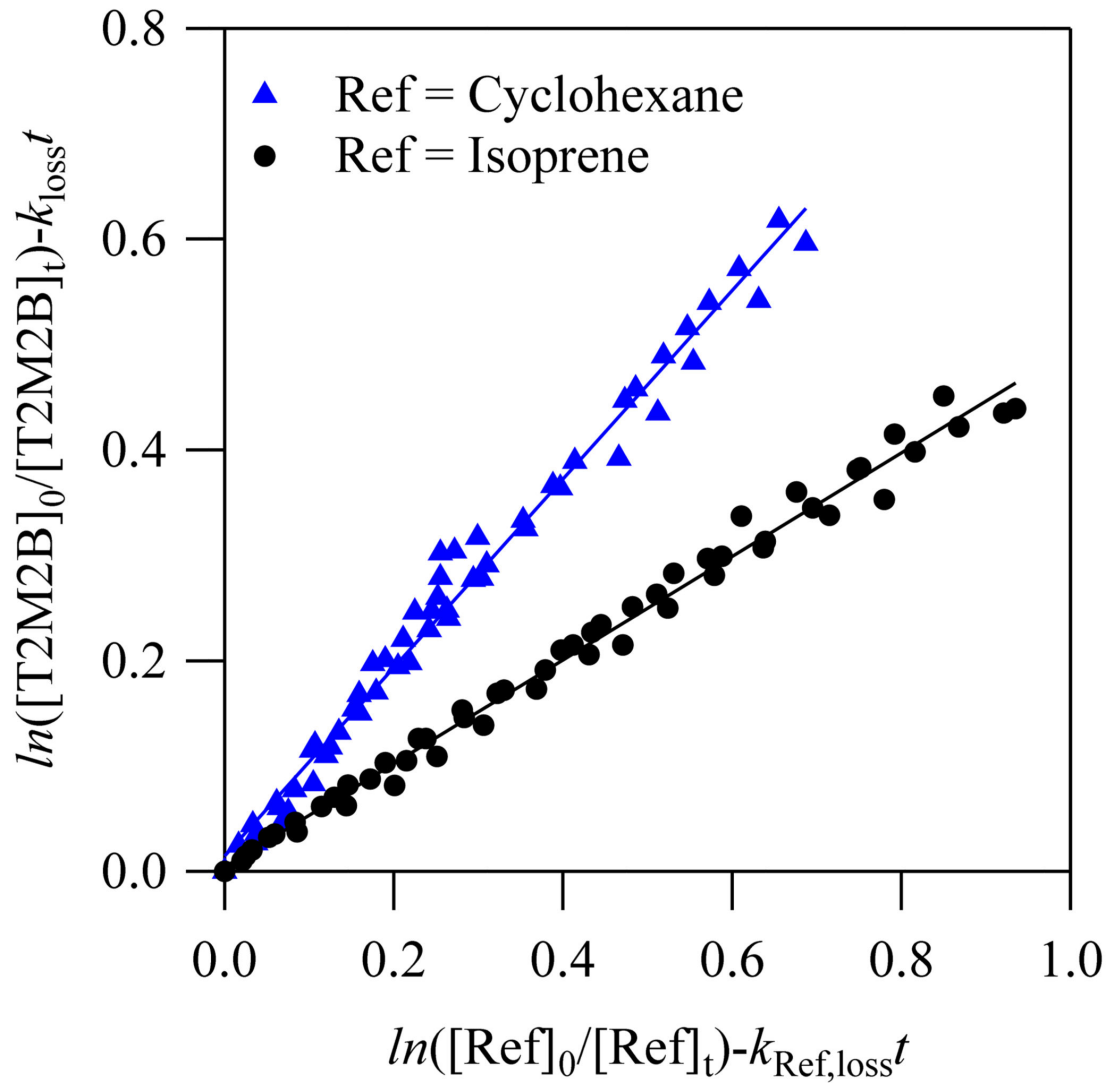
Plot of Equation (1) for the two references used in this research.

In Figure 1, the loss of T2M2B with respect to that for the reference compound is plotted for all the experiment series carried out with each reference compound. Plots of ln([T2M2B]_0_/[T2M2B]_t_)-k_loss_ *t* versus *ln*[Ref]_0_/[Ref]_t_)-*k*_Ref,loss_ *t* show a good linearity, indicating that there were no kinetic complications. According to Equation (1), *k*/*k*_Ref_ was obtained from the slope of these plots. Then, the rate coefficient of reaction (R1), *k*, was determined at room temperature considering the rate coefficients of the reference compounds previously reported for the Cl reaction of cyclohexane and isoprene [21,22]. The individual rate coefficients k are summarized in Table 2 for the two reference compounds. The averaged value of k was determined to be (2.45 ± 0.32) × 10^−10^ cm^3^ molecule^−1^ s^−1^. The ±2σ uncertainty in *k* includes the propagation of the reported errors in *k*_Ref_, the statistical errors from the slope of the plots shown in Figure 1, and the uncertainties in *k*_loss_.

### 3.2 Identification of the Reaction Products in the Gas-Phase

As mentioned in the previous section, complementary detection techniques were used to identify and/or quantify the products generated in reaction (R1): SPME/GC-MS, FTIR spectroscopy, and PTR-ToF-MS. Prior to that, tests were done to check if products were generated during the UV light exposure of T2M2B and its dark reaction with Cl_2_ In the experiments performed with SPME/GC-MS, no products were observed in these tests, whereas when PTR-ToF-MS and FTIR spectroscopy were used, it was possible to detect the formation of some products during the UV light exposure of T2M2B.

#### 3.2.1 Detection by SPME/GC-MS

**Figure 2:**
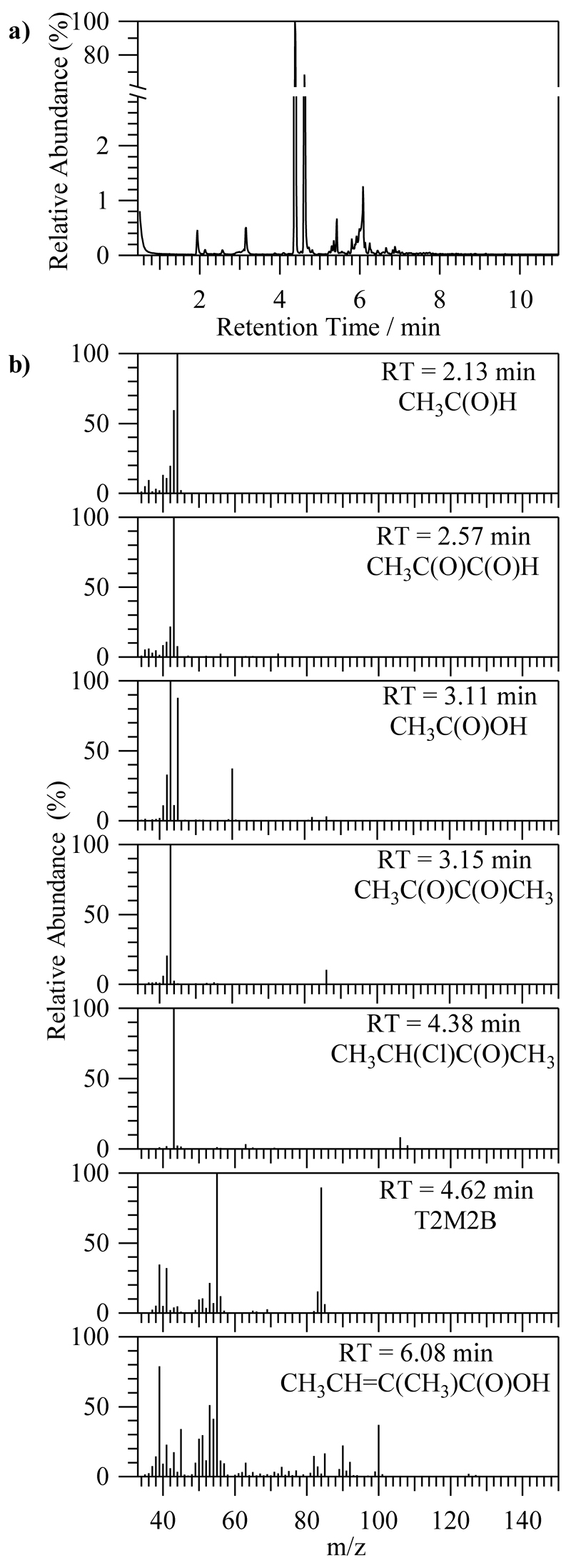
(**a**) Chromatogram obtained after 60 min of the chlorine (Cl) reaction of *trans*-2-methyl-2-butenal (T2M2B). (**b**) Mass spectra of the most intense peaks of the chromatogram that were attributed to products.

Figure 2a shows the chromatogram obtained after 60 min of irradiation. The peak corresponding to T2M2B was observed at a retention time (RT) of 4.62 min. The new peaks that appeared in the chromatogram can be assigned to a product according to the mass spectrum of each RT (in min), as shown in Figure 2b. The detected products were acetaldehyde (RT = 2.13), methylglyoxal (RT = 2.57), acetic acid (RT = 3.11), 2,3-butanedione (RT = 3.15), 3-chloro-2-butanone (RT = 4.38, most intense), and 2-methyl-2-butenoic acid (RT = 6.08). Other small peaks in the chromatogram were due to the degradation of the SPME fiber Atmosphere 2020, 11, x FOR PEER REVaIEnWd the column.

#### 3.2.2 Detection by PTR-ToF-MS

**Figure 3:**
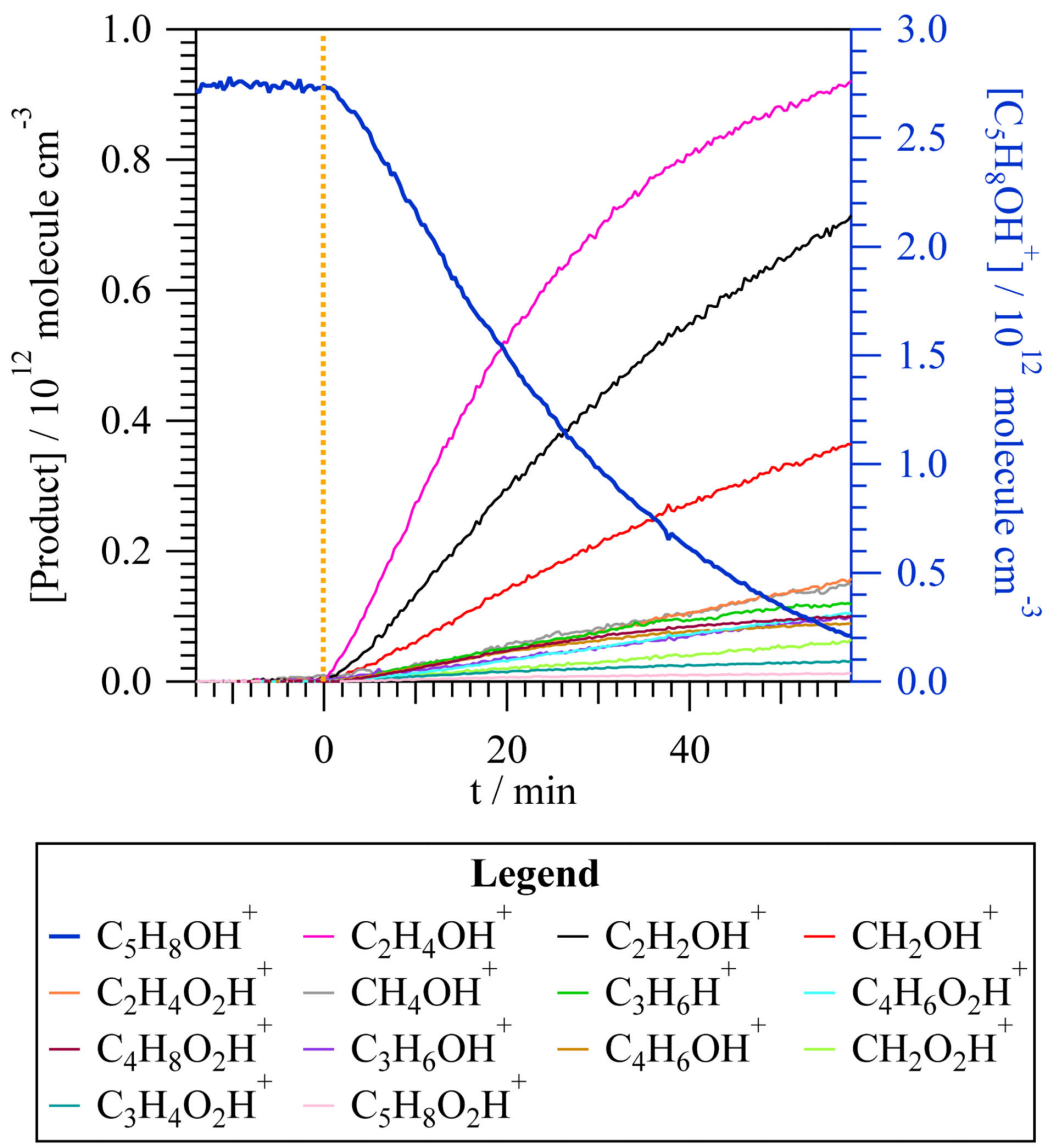
Time evolution of the concentration of T2M2B (C_5_H_8_OH^+^, on the right axis) and the products measured by proton transfer time-of-flight mass spectrometry (PTR-ToF-MS) during the *trans*-2-methyl-2-butenal + Cl reaction. *t* = 0 min, depicted with a yellow dashed line, was set as the moment in which the reaction started when the lamps were turned on.

The PTR-ToF-MS detects protonated molecular ions for non-chlorinated organic compounds. All the reaction products observed by SPME/GC-MS, except 3-chloro-2-butanone, were also observed by PTR-ToF-MS: acetaldehyde (C_2_H_4_OH^+^, *m*/*z* = 45.03), acetic acid (C_2_H_4_O_2_H^+^, *m*/*z* = 61.02), methylglyoxal (C_3_H_4_O_2_H^+^, *m*/*z* = 73.02), 2,3-butanedione (C_4_H_6_O_2_H^+^, *m*/*z* = 87.05), and 2-methyl-2- butenoic acid (C_5_H_8_O_2_H^+^, *m*/*z* = 101.6). In addition, other products were observed such as formaldehyde (CH_2_OH^+^, *m*/*z* = 31.01), methanol (CH_4_OH^+^, *m*/*z* = 33.03), ketene (C_2_H_2_OH^+^, *m*/*z* = 43.01) propene (C_3_H_6_H^+^, *m/z* = 43.05), formic acid (CH_2_O_2_H^+^, *m/z* = 47.01), propanal (C_3_H_6_OH^+^, *m/z* = 59.08), butyric acid (C_4_H_8_O_2_H^+^, *m/z* = 89.05), and 2-butenal (C_4_H_6_OH^+^, *m/z* = 71.03). Given that molecular ions are detected, it is possible that an isomer of the mentioned products would be present. In Figure 3, it is observed that the major products detected by PTR-ToF-MS are acetaldehyde, ketene, and formaldehyde, with product yields of (40.8 ± 0.6)%, (23.5 ± 0.4)%, and (11.0 ± 0.2)%, respectively. For the minor reaction products, the yields were determined to be less than 5%. The three major products, acetaldehyde, ketene, and formaldehyde, were also formed during the exposure of T2M2B to the UV light in the test experiments performed prior the Cl reaction, but their amount was negligible compared with the observed concentration after the Cl reaction.

#### 3.2.3 Detection by FTIR Spectroscopy

**Figure 4:**
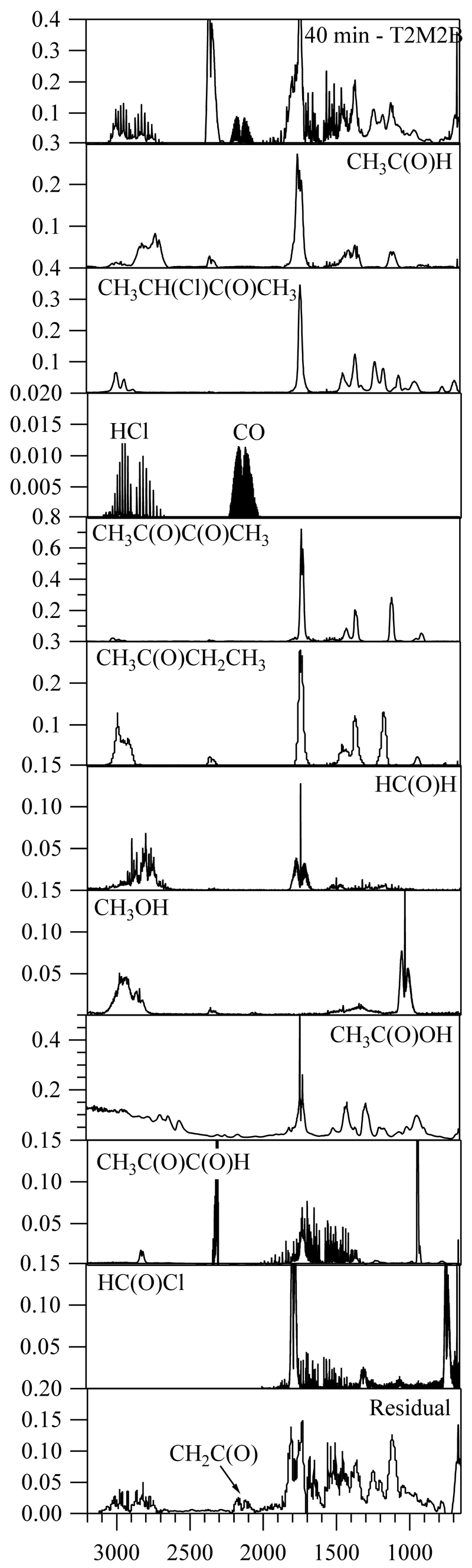
FTIR spectra. Top panel shows the spectrum obtained after 40 min of Cl reaction of *trans*-2- methyl-2-butenal with the features of this compound subtracted. The bottom panel shows the residual spectrum after the subtraction of the IR features corresponding to the detected products. HC(O)Cl spectrum was provided by Nielsen [24] and the CH_3_C(O)C(O)H spectrum was taken from the FTIR database of the EUROCHAMP project [25].

The FTIR spectra recorded during reaction (R1) showed features that agree with the products observed by SPME/GC-MS and PTR-ToF-MS as can be seen in Figure 4. By comparison with the reference spectra (shown in central panels of Figure the most abundant products observed were HCl, CO, 3-chloro-2-butanone, and acetaldehyde. The obtained product yields were Y_HCl_ = (92.8 ± 12.9)%, Y_CO_ = (97.3 ± 10.0)%, and Y_3-chloro-2-butanone_ = (52.5 ± 7.3)%. The quantification of acetaldehyde by FTIR spectroscopy was very imprecise due to the presence of other minor products that have similar features in the IR spectrum. The minor products that were identified are formaldehyde, acetyl chloride, methanol, methyl glyoxal, acetic acid, 2,3-butanedione, and butanone. After subtracting all these products and by comparison with the IR features described by Wallington et al. [23], ketene (CH_2_C(O)) was also identified in the 2100–2200 cm^−1^ range of the residual spectrum. No reference spectrum was available for ketene given its instability. Despite this identification, there are still some IR features that could be due to an unknown carbonyl compound or 2-methyl-2-butenoic acid. This acid was observed by SPME/GC-MS and PTR-ToF-MS, but no IR reference spectrum could be obtained due to experimental limitations. Nevertheless, given the amount detected by the two other techniques, it is expected that the concentration detected by FTIR will be very low too. During the exposure to UV light test of T2M2B, small amounts of CO, formaldehyde, and acetaldehyde were detected, but, similarly, as observed by PTR-ToF-MS, in a lesser extent than the observed during the Cl reaction.

### 3.3 SOA Formation Study

**Figure 5:**
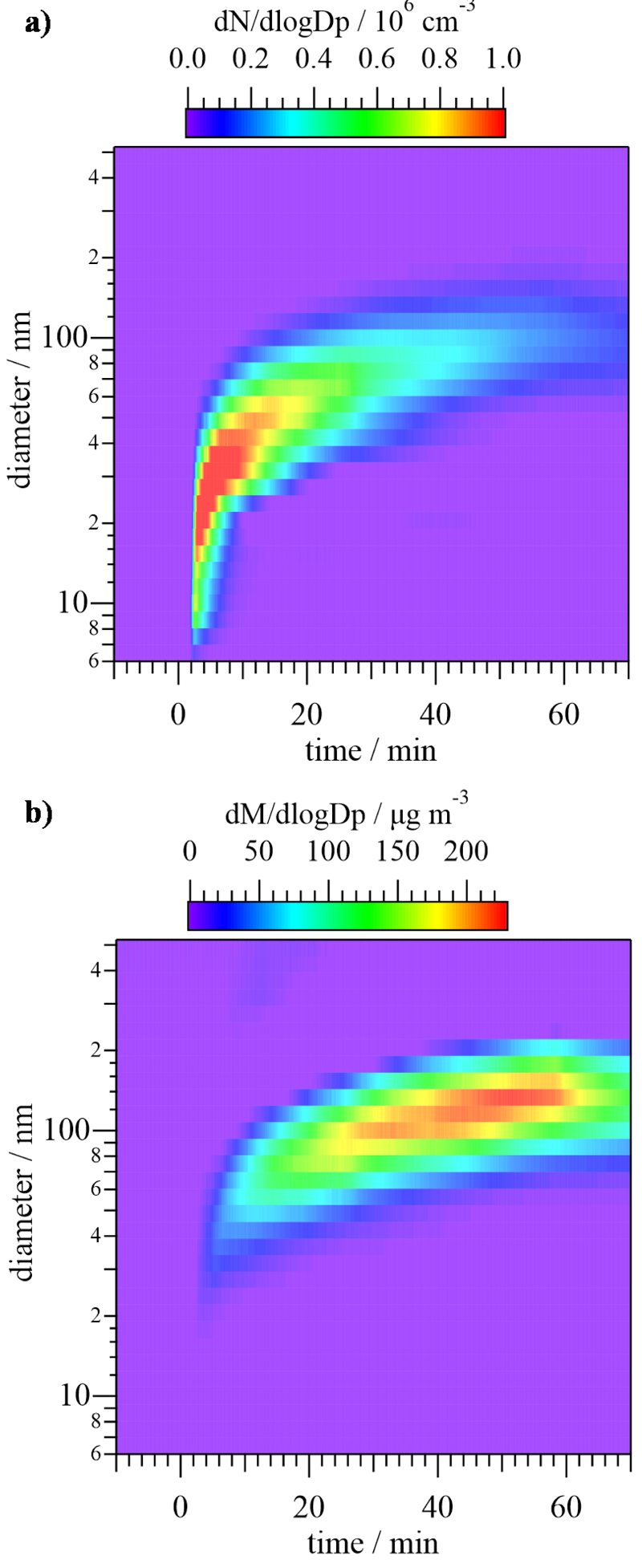
Evolution of the size of the secondary organic aerosol (SOA) generated in the Cl reaction of T2M2B in terms of the normalized particle number (**a**) and mass (**b**). Reaction starts at *t* = 0 min and ends at *t* = 60 min.

In Figure 5, the size distribution of particles is shown in terms of the normalized particle number, *d*N/*d*logDp, and mass, *d*M/*d*logDp. As depicted in Figure 5a, shortly after the beginning of the Cl reaction (at *t* = 2 min), the appearance of a high concentration (in terms of number) of ca. 20-nm particles was observed. At subsequent times, the diameter of the particles increased, being centered at ca. 100 nm at *t* = 60 min, while the number of particles decreased. Figure 5b shows that the maximum mass of the first formed particles was centered at a ca. 30 nm diameter, reaching a value of around 10 μg m^−3^. As Cl reaction continued, the maximum of the mass increased up to 200 μg m^−3^ and was centered at 130 nm. Both graphs indicate that the formation of a big number of small particles took place 2 min after Cl reaction started and this formation was due to a nucleation process. It was followed by the growth of the particles due to coagulation, which caused that the number of particles was reduced at longer times even though the mass kept increasing.

**Figure 6:**
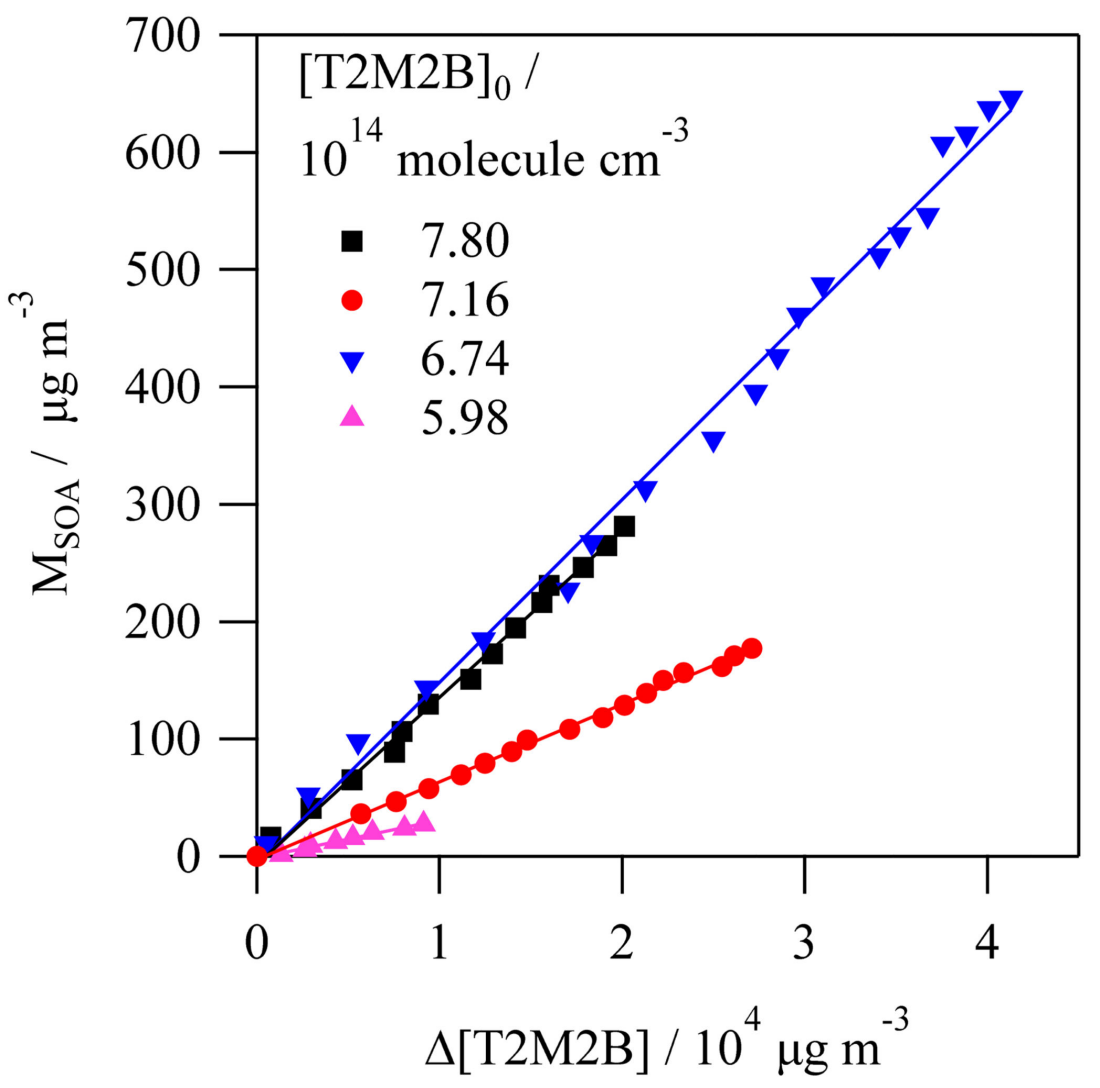
Some examples of the plots of SOA mass (M_SOA_) versus Δ[T2M2B] used to determine the SOA yield (Y_SOA_).

The SOA yield, Y_SOA_, was determined under different conditions from the slope of the plot of M_SOA_ versus Δ[T2M2B] (Figure 6). The correction of M_SOA_ was done considering the rate coefficient for the loss of particles determined in each experiment (average value: *k*_loss particle_ = 4.2 × 10^−2^ s^−1^). The rate coefficient for the dark loss of T2M2B was also determined at the beginning of each experiment and was used for correcting its concentration at each reaction time. This rate coefficient averaged *k*_loss_ = 3.4 × 10^−3^ s^−1^. The results of Y_SOA_ are listed in Table 3 together with the conditions used in this research.

**Figure 7:**
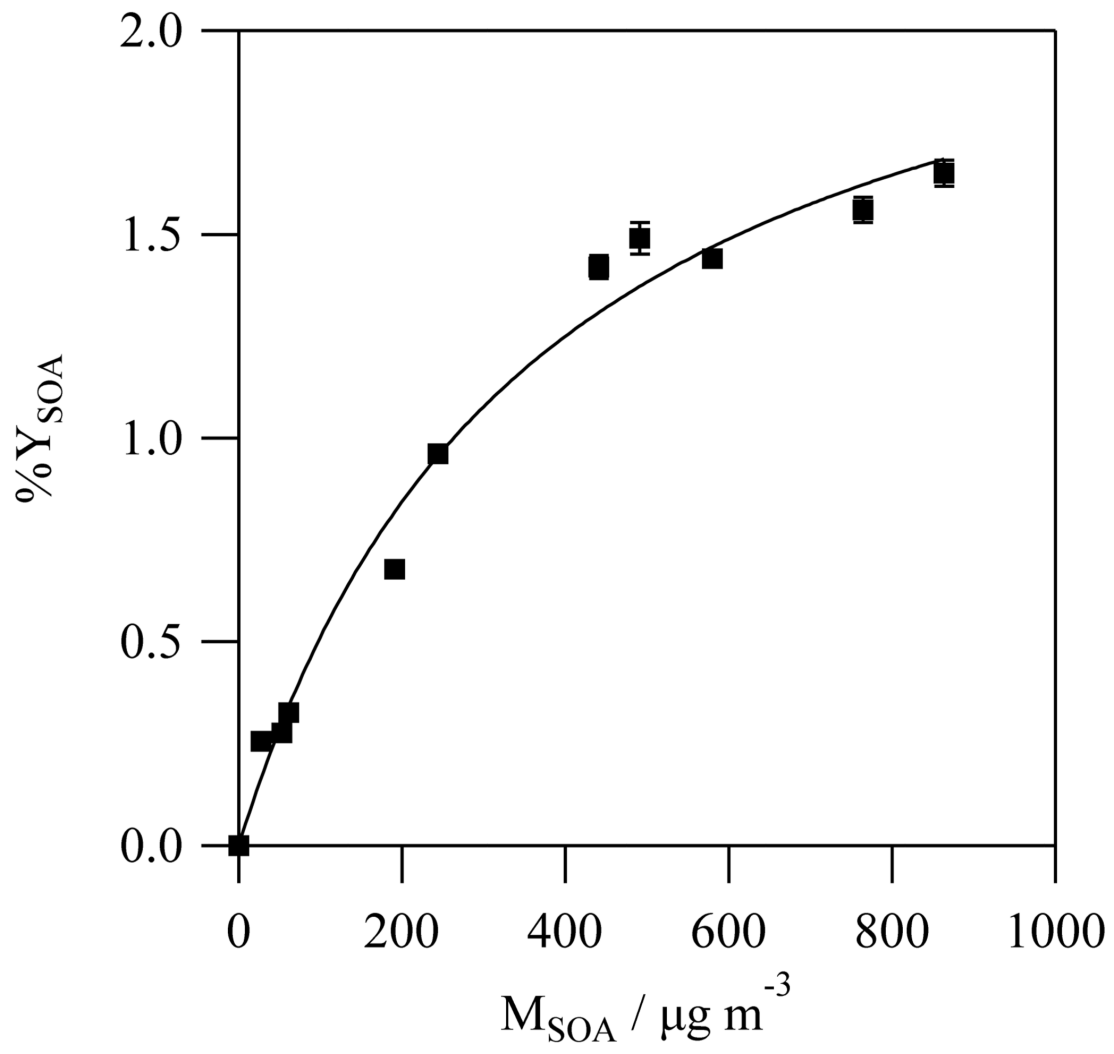
SOA yield curve determined in this research for the *trans*-2-methyl-2-butenal reaction with Cl.

The gas/particle absorption model proposed by Pankow [26,27] can describe the mechanism of SOA formation accordingly to the relationship between Y_SOA_ and the maximum value of M_SOA_ reached in every experiment [28]:

(5) $${\text{Y}}_{\text{SOA}}={\text{M}}_{\text{SOA}}\sum_{\text{i}}^{\text{n}}\frac{\alpha_{i}K_{p,i}}{1+K_{p,i}{\text{M}}_{\text{SOA}}},$$

where *α_i_* is the mass-based stoichiometric coefficient and *K*_p,*i*_ is the gas/particle partitioning coefficient of the *i* semi-volatile species formed. In this research, data could be fitted to a one-product model (*i* = 1), as can be seen in Figure 7, yielding α = (2.4 ± 0.2)%, and *K*_p_ = (2.7 ± 0.5) × 10^−3^ m^3^ μg^−1^. These parameters indicate, respectively, that Y_SOA_ tends towards 2.4% at very high M_SOA_ values and that the semi-volatile product that causes the SOA formation is mostly in the vapor phase rather than in the condensed phase.

## 4 Discussion

### 4.1 Comparison of the Cl Reactivity with Unsaturated Aldehydes

As far as we know, the present research is the first kinetic study of the reaction Cl + T2M2B. The rate coefficient that has been determined in this study is within the same order of magnitude of the reported for other aldehydes as summarized in Table 4. The rate coefficients for the other aldehydes were determined by using a relative method in all cases, similarly to this research, with the techniques shown in Table 4.

Considering the available kinetic data for the Cl reaction with other unsaturated aldehydes, a structure–activity trend of the Cl reactivity can be derived. As shown in Table 4 and also reported by Rodríguez et al. [9], the rate coefficient seems to slightly increase with the hydrocarbon chain for the linear unsaturated aldehydes, *E*-CH_3_(CH_2_)_x_CH=CHC(O)H, when x = 5–7. This would indicate that the H-abstraction from the hydrocarbon chain is playing a role. However, the rate coefficient determined for a shorter unsaturated aldehyde, *E*-CH_3_CH=CHC(O)H, reported by Thévenet et al. [10], indicates that this trend may not be as clear and further studies would be needed. For branched unsaturated aldehydes with a central double bond, such as *E*-CH_3_CH=C(CH_3_)C(O)H and (CH_3_)_2_C=CHC(O)H, the Cl reactivity seems to increase, according to what has been reported by Rodríguez et al. [10], with respect to that of the linear unsaturated aldehyde E-CH_3_CH_2_CH=CHC(O)H, that has the same number of C andHatoms. This trend could indicate that the branching of the main chain by the electron donor methyl groups accelerates the reaction. However, the rate coefficient for E-CH_3_CH=CHC(O)H, reported by Thévenet et al. [10], is similar to the reported in this research for E-CH_3_CH=C(CH_3_)C(O)H, which would indicate that the presence of the methyl group has no effect on the reactivity. As stated above, further studies on the reactivity of the linear unsaturated aldehydes are needed to better establish the reactivity trend as a function of the structure. When looking at the length of the chain of branched unsaturated aldehydes, it seems to slightly reduce the Cl reactivity as observed when comparing the rate coefficients for T_2_M_2_B and methacrolein (CH_2_=C(CH_3_)C(O)H) [11]. It is also possible to compare the effect of the position of the methyl group in the reactivity by looking at the rate coefficients of 3-methyl-2-butenal, (CH_3_)_2_C=CHC(O)H, [12], and T2M2B. Both compounds have almost the same rate coefficient (considering the errors), indicating that the position of the methyl group does not affect the Cl reactivity in this case. Finally, the Cl-reactivity of saturated aldehydes was reported to be faster than that for unsaturated aldehydes [9]. There are no available data for the reaction of 2-methylbutanal with Cl to compare with trans-2-methyl-2-butenal, and thus the comparison can be done with pentanal, CH_3_(CH_2_)_3_C(O)H, which has the same amount of C atoms, showing a very similar rate coefficient ((2.56 ± 0.27) × 10^−10^ cm^3^ molecule^−1^ s^−1^, [9]).

### 4.2 Reaction Mechanism to Form Gaseous Products

**Figure 8:**
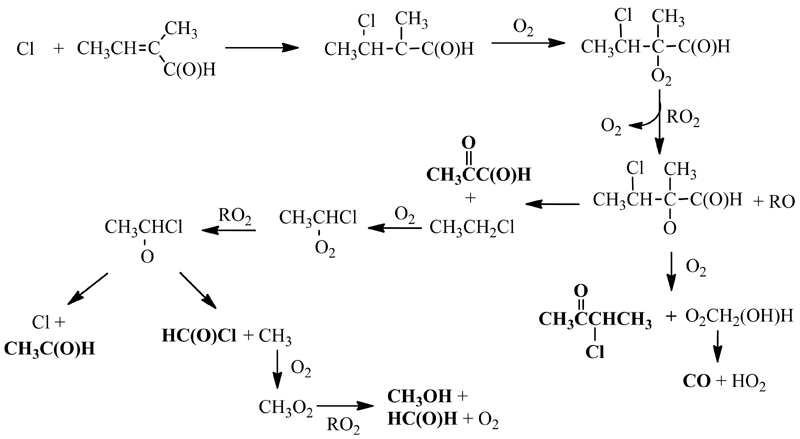
Proposed mechanism for the Cl reaction of *trans*-2-methyl-2-butenal through for the Claddition to C-3.

**Figure 9:**
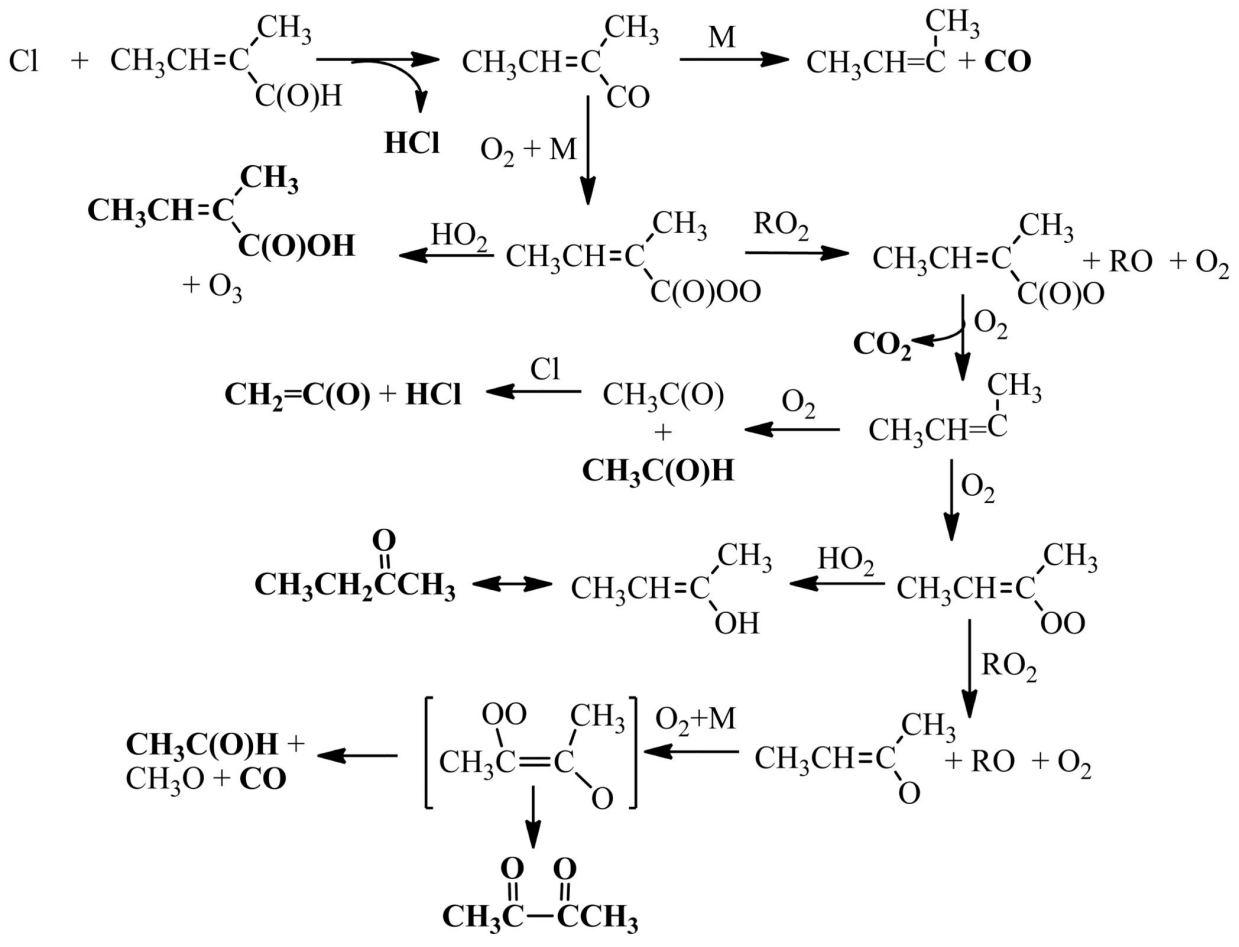
Proposed mechanism for the Cl reaction of *trans*-2-methyl-2-butenal through the initial Habstraction from the aldehyde group.

The great variety of products detected by three complementary techniques allows to propose a reaction mechanism. What seems clear is that HCl, CO, 3-chloro-2-butanone, and acetaldehyde are the products for which the yields are higher. There are two reaction pathways, depicted in Figures 8 and 9, that can explain the presence of these reaction products: the addition of Cl to the double bond in the C-3 position (Figure 8) and the H-abstraction from the aldehyde group (Figure 9). In both figures, the species in bold are the ones identified in this study.

The addition of Cl to C-3 of T2M2B can explain the formation of the observed products: 3-chloro-butanone, methylglyoxal, acetaldehyde, acetyl chloride, methanol, and formaldehyde. The initial step of this pathway leads to the formation of a chlorinated alkyl radical (CH_3_CH(Cl)C(CH_3_)C(O)H) that, following the general reactivity trend, reacts with O_2_ and a peroxy radical to generate the alkoxy radical CH_3_CH(Cl)CO(CH_3_)C(O)H. This alkoxy radical can react with O_2_, as described by Hasson et al. [29] for carbonyl containing peroxy radicals, to form 3-chloro-2-butanone, CH_3_CH(Cl)C(O)CH_3_, or it can decompose to generate methylglyoxal (CH_3_C(O)C(O)H)) and the CH_2_CHCl radical that continues to react with O_2_, RO_2_, and decompose to generate acetaldehyde (CH_3_C(O)H) and acetyl chloride (HC(O)Cl), and, through further reactions of the methyl radical, methanol (CH_3_OH) and formaldehyde (HC(O)H). However, the decomposition route of the CH_3_CH_2_Cl radical seems to be almost negligible, given the low yield of methyl glyoxal, (1.17 ± 0.08)%. The C-3 addition mechanism was expected, according to previous studies for unsaturated aldehydes [9], to be inhibited because of the conjugation between the double bond and the carbonyl group that reduces the partial negative charge on that carbon atom. However, the presence of the methyl group in this molecule seems to activate this channel, since one of the most abundant products observed, 3-chloro-2-butanone (CH_3_CH(Cl)C(O)CH_3_) can only be explained by this reaction pathway. The observed yield for this product indicates that this pathway represents around 53% of the overall reaction.

The H-abstraction from the -C(O)H group can explain the presence of acetaldehyde, HCl, CO, 2-methyl-2-butenoic acid, butanone, butadiene, and ketene. The first step leads to the formation of HCl, a major product, and a carbonyl radical that can decompose or react with O_2_ followed by the reaction with RO_2_ or HO_2_ radicals. The reaction with HO_2_ can generate the 2-methyl-2-butenoic acid, CH_3_CH=C(CH_3_)C(O)OH, one of the minor products of this reaction, the decomposition generates the CH_3_CH=CCH_3_ radical, and the RO_2_ reaction leads to the formation of the CH_3_CH=C(CH_3_)C(O)O radical that can decompose to generate CO_2_ and the unsaturated alkyl radical CH_3_CH=CCH_3_, as described in various previous studies for unsaturated aldehydes [30–32]. The CH_3_CH=CCH_3_ radical reacts with O_2_ to form acetaldehyde, CH_3_C(O)H, and the acetyl radical, CH_3_C(O), or the CH_3_CH=C(CH_3_)OO radical. The acetyl radical can react with Cl atoms and can generate HCl and ketene [33], another product observed at high concentration. The CH_3_CH=C(CH_3_)OO radical can react either with HO_2_ or RO_2_ radicals. The reaction with HO_2_ radicals explains the generation of butanone (CH_3_CH_2_C(O)CH_3_) through its corresponding enol. The RO_2_ reaction followed by the reaction with O_2_ generates an intermediate similar to what has been previously suggested by Magneron et al. [30] and which can decompose to form CO and acetaldehyde, or can isomerize to 2,3-butanedione (CH_3_C(O)C(O)CH_3_).

The addition of the Cl atom to C-3 of T2M2B was also considered as possible, since acetaldehyde, formaldehyde, and CO could also be generated through this pathway. However, the presence of other chlorinated products such as CH_3_CH(Cl)C(O)H or ClC(O)CH_3_, that would be generated if this mechanism was occurring, could not be confirmed with the analytical techniques used in this research, so this pathway seems to be negligible. Given that acetaldehyde is generated mostly by the H-abstraction pathway, its molar yield shows that around 40% of the overall reaction proceeds through this pathway, as depicted in Figure 9. Theoretical calculations on the relative importance of the reaction pathways of the first step of the mechanism could be useful to confirm the reaction pathways reported in this study.

### 4.3 SOA Formation Study

The SOA yields determined in this research for the Cl reaction of T2M2B range between 0.26% and 1.65% under NO_x_-free conditions. Under high NO_x_ conditions and using ammonium sulfate as seed, Chan et al. reported a much higher SOA yield (39.1%) for the corresponding OH reaction [18]. The difference between the two studies may be due to the formation of less volatile products in the OH reaction than those obtained in the Cl reaction that contribute to the SOA formation. However, there are studies that show that SOA yields in the Cl reactions are comparable to the formed in other oxidation scenarios (OH or O_3_ reaction, photolysis, etc.), such as a study reported for some monoterpenes [34]. Therefore, the low values determined in this research compared to the OHreaction [18] can beattributed to the different conditions that were used and that may change the mechanism of SOA formation and affecting the SOA yield. First, the effect of NO_x_ in Y_SOA_ seems to be very complex as there are multiple studies that show contradictory results, as summarized by Chan et al. [18], so it is hard to state clearly if the observed difference can be due to this factor. Secondly, the presence of ammonium sulfate seeds enhances Y_SOA_ [35] and would explain the difference observed between the OH reaction studied by Chan et al. [18] and the results presented in this study. Moreover, there are other factors that can affect the SOA yields, such as the temperature and the relative humidity. Temperature in this research was slightly higher than the temperature set by Chan et al. [18] (the difference is less than 10 K), and at higher temperatures the semi-volatile species are more present in the gas phase, but it is hard to know if this can explain the lower SOA yields observed in this study. Finally, the relative humidity in both studies is similar, since no additional H_2_O was added in any of them, so this factor should not be the source of the difference observed.

### 4.4 Atmospheric Implications

The tropospheric lifetime due to the Cl reaction of *trans*-2-methyl-2-butenal, τ_Cl_, was estimated as in previous studies [19,20] using the rate coefficient determined in this research and two different Cl atoms concentrations: a 24 h average (1 × 10^3^ atom cm^−3^, [36]) and a peak concentration (1.3 × 10^5^ atom cm^−3^, [37]). The reason for this is to evaluate the influence of this reaction in areas with low levels of Cl and other zones with high Cl concentration, such as coastal or industrial areas. In the first scenario, τ_Cl,24h_ was estimated to be 46 days, while considering the peak concentration, τ_Cl,peak_ was 8.7 h. The individual tropospheric lifetimes for the OH reaction and the O_3_ reaction of T2M2B were previously estimated as 6.8 h [13] and 3 days [14], respectively. Therefore, if the peak Cl scenario is considered, the tropospheric lifetime due to the three homogeneous reactions is 3.6 h. In this case the contribution of the Cl reaction to the T2M2B homogeneous loss is 42%, very similar to the OH radical contribution (53%). This indicates that, although the Cl reaction is faster than the OH reaction by one order of magnitude, the importance of this degradation route in the troposphere can be approximately the same. The upper limit of the tropospheric lifetime due to UV photolysis was estimated in a previous study as 0.7 h [38], thus, in the most favorable conditions for photolysis this would be the most important degradation route.

In terms of the contribution of the Cl + T2M2B reaction to the formation of secondary pollutants, 3-chloro-2-butanone and acetaldehyde can be formed with 52.5% and 40.8% yields, respectively, contributing to smog episodes in clean atmospheres where low NO_X_ levels are present. Further photooxidation of these carbonyl compounds can also yield to other pollutants. The concentration levels of these species and, therefore, their atmospheric impact will depend on the emitted quantity of T2M2B. As far as we know, this compound has not been detected at high concentrations in the troposphere, so the impact of the generated products is expected not to be very important. The contribution of the SOA formed in the Cl + T2M2B reaction can be considered as negligible taking into account that the yield is Y_SOA_ < 2.4%, so its impact on the air quality is expected to be low. Finally, as HCl and CO are also formed with 92.8% and 97.3% yields, respectively, they can impact at a local or regional scale contributing to the acidity of the atmosphere [39] and the formation of the tropospheric ozone in a polluted environment, respectively.

## 5 Conclusions

In this research, we have reported for the first time the room temperature rate coefficient of the reaction between Cl and *trans*-2-methyl-2-butenal at 760 ± 5 Torr. Although this rate coefficient is quite high, with a similar value as the previously reported rate coefficients for other saturated and unsaturated aldehydes, the contribution of the Cl reaction to the homogeneous loss of T2M2B in the troposphere is less than 42%. This research also presents the first detailed product study for the titled reaction, using three complementary detection techniques. In summary, the main gaseous products identified were

- Acetaldehyde, methylglyoxal, acetic acid, and 2,3-butanedione, detected by the three techniques;
- 3-chloro-2-butanone, detected by SPME/GC-MS and FTIR;
- Formaldehyde, methanol, and ketene, detected by PTR-ToF-MS and FTIR;
- HCl, CO, and ClC(O)H, detected only by FTIR.

The major reaction products are HCl ((92.8 ± 12.9)%), CO ((97.3 ± 10.0)%), 3-chloro-2-butanone ((52.5 ± 7.3)%), and acetaldehyde ((40.8 ± 0.6)%), indicating that the Cl-addition to C-3 and the H-abstraction from the aldehyde group of T2M2B are the major reaction pathways with around 53% and 40% contribution, respectively. Formation of ultrafine particles (5.6–560 nm) was investigated for the first time and we concluded that the SOA yield is very low. In conclusion, the impact of the observed degradation products, especially secondary pollutants (HCl, CO, carbonyl compounds, and aerosols) on air quality or acid rain episodes is expected to be low at the expected mission levels of T2M2B.

## Figures and Tables

**Table 1:** Rate coefficients for the different loss processes observed for the compounds used in the kinetic study. Errors are ±2σ.

Compound	k_w_/10^−6^ s^−1^	k_hν_/10^−4^ s^−1^	k_Cl_2__/10^−20^ cm^3^ molecule^−1^ s^−1^
*Trans*-2-methyl-2-butenal	8.40 ± 5.33	8.33 ± 5.60	-
Cyclohexane	0.400 ± 0.417	-	-
Isoprene	3.78 ± 1.65	-	2.70 ± 0.15

**Table 2:** Results obtained in the kinetic experiments for reaction (R1). Errors are e ± 2σ.

Reference	*k*/*k*_Ref_	*k*_Ref_/10^−10^ cm^3^ molecule^−1^s^−1^	*k*/10^−10^ cm^3^ molecule^−1^s^−1^
Cyclohexane	0.894 ± 0.030	3.08 ± 0.12^1^	2.75 ± 0.14
Isoprene	0.491 ± 0.014	4.35 ± 0.58^2^	2.14 ± 0.29
Averge			2.45 ± 0.32

1 Aschmann and Atkinson [21].

2 Orlando et al. [22].

**Table 3:** Results of Y_SOA_ under different conditions. Errors are ±2σ.

[T2M2B]_0_/10^14^ molecule cm^−3^	[Cl_2_]_0_/10^14^ molecule cm^−3^	M_SOA_/μgm^−3^	Y_SOA_(%)
5.62	4.46	27.4	0.26±0.01
6.79	2.54	52.9	0.27±0.01
5.99	2.67	60.9	0.33±0.01
7.17	5.96	191	0.68±0.01
6.31	6.93	244	0.96±0.01
7.79	9.53	441	1.42±0.03
7.58	12.6	491	1.49±0.04
7.39	15.0	580	1.44±0.02
6.74	17.0	764	1.56±0.03
7.10	19.9	863	1.65±0.03

**Table 4:** Summary of the Cl-rate coefficients of selected unsaturated aldehydes at 298 K.

Unsaturated Aldehyde	*k*/10^−10^ cm^3^ molecule^−1^ s^−1^	Technique	Reference
Linear	*E*-CH_3_CH=CHC(O)H	2.60 ± 0.04	GC-FID	[10]
	*E*-CH_3_CH_2_CH=CHC(O)H	1.31 ± 0.19	GC-MS	[9]
	*E*-CH_3_(CH_2_)_2_CH=CHC(O)H	1.92 ± 0.22	GC-MS	[9]
	*E*-CH_3_(CH_2_)_3_CH=CHC(O)H	2.40 ± 0.29	GC-MS	[9]
Branched	*E*-CH_3_CH=C(CH_3_)C(O)H	2.45 ± 0.32	FTIR	This research
	(CH_3_)_2_C=CHC(O)H	2.48 ± 0.71	FTIR	[12]
	CH_2_=C(CH_3_)C(O)H	2.9 ± 0.8	FTIR and GC-FID	[11]
